# FindICI: Using machine learning to detect linguistic inconsistencies between code and natural language descriptions in infrastructure-as-code

**DOI:** 10.1007/s10664-022-10215-5

**Published:** 2022-09-20

**Authors:** Nemania Borovits, Indika Kumara, Dario Di Nucci, Parvathy Krishnan, Stefano Dalla Palma, Fabio Palomba, Damian A. Tamburri, Willem-Jan van den Heuvel

**Affiliations:** 1 Jheronimus Academy of Data Science, Tilburg University, Tilburg, The Netherlands; 2 University of Salerno, Salerno, Italy; 3 Jheronimus Academy of Data Science, Technical University Eindhoven, Eindhoven, The Netherlands

**Keywords:** Infrastructure as code, Linguistic anti-patterns, Word embedding, Machine learning, Deep learning

## Abstract

Linguistic anti-patterns are recurring poor practices concerning inconsistencies in the naming, documentation, and implementation of an entity. They impede the readability, understandability, and maintainability of source code. This paper attempts to detect linguistic anti-patterns in Infrastructure-as-Code (IaC) scripts used to provision and manage computing environments. In particular, we consider inconsistencies between the logic/body of IaC code units and their short text names. To this end, we propose FindICI a novel automated approach that employs word embedding and classification algorithms. We build and use the abstract syntax tree of IaC code units to create code embeddings used by machine learning techniques to detect inconsistent IaC code units. We evaluated our approach with two experiments on Ansible tasks systematically extracted from open source repositories for various word embedding models and classification algorithms. Classical machine learning models and novel deep learning models with different word embedding methods showed comparable and satisfactory results in detecting inconsistent Ansible tasks related to the top-10 used Ansible modules.

## Introduction

The software development cycle is becoming shorter every day. Therefore, development and IT operation teams are increasingly cooperating as DevOps teams, relying massively on automation at both development and operations levels. The software code driving such automation is collectively known as Infrastructure-as-Code (IaC), a model for provisioning and managing a computing environment using the explicit definition of the desired state of the environment in source code and applying software engineering principles, methodologies, and tools (Morris 2016).

Although IaC is a relatively new research area, it attracted an ever-increasing number of scientific works in recent years (Rahman et al. 2019). Although most research on IaC investigated its tools, adoption, and testing (Rahman et al. 2019), only a few studies explored its code quality. The first steps in this direction focused on applying the well-known concept of software defect prediction (Hall et al. 2011) to infrastructure code defining defect prediction models (Rahman and Williams^2018^, 2019a; Dalla Palma et al. 2021) to identify pieces of infrastructure that may be defect-prone and need more inspection. In this perspective, previous works mainly focused on the identification of structural code properties that correlate with defective infrastructure code scripts and the detection of various smells. However, defects are only a possible issue given that problems with the source code lexicon can negatively affect code comprehensibility and maintainability (Lawrie et al. 2007; Takang et al. 1996). Linguistic anti-patterns are common code lexicon problems, i.e., recurring poor practices concerning inconsistencies between the naming, documentation, and implementation of entities (Arnaoudova et al. 2016,2013) Linguistics anti-patterns can also be exhibited in IaC programs. While the existing literature mainly focuses on structural characteristics of defective IaC scripts, none exists that analyze linguistic issues to the best of our knowledge. This motivation led to the research goal of this work:

*Can we accurately detect mismatches between IaC code units and their short natural language descriptions using a learning-based approach?*

Boosted by the emerging trend of learning-based approaches and word embedding in the software engineering research (Sulistya et al. 2020; Pradel and Sen 2018; Liu et al 2019; Omri and Sinz 2020; Li et al. 2020), we propose FindICI, a novel approach to detect linguistic anti-patterns in IaC, focusing on name-body inconsistencies in IaC code units. We formulate name-body inconsistency detection as a binary classification problem and train a classifier that distinguishes between consistent and inconsistent code units. We use Ansible as the IaC language, which is one of the widely-used IaC languages (Guerriero et al. 2019), where a task is a unit of provisioning and configuration logic. A task has a name and a body. The task name is essentially a short text that communicates the purpose of the task. Our approach leverages the word embedding models to produce distributed representations (feature vectors for the classifiers) of task names and bodies, respectively. We evaluated the effectiveness of our approach on a dataset composed of Ansible tasks for the top 10 used Ansible modules from 38 open source repositories using machine learning and neural networks trained using different word embedding representations.

Our experiments show that various learning algorithms can successfully detect inconsistent IaC code units with high performance in MCC, AUC-ROC, and accuracy. Similarly, all word embedding models also showed good performance in terms of the evaluation metrics MCC, AUC-ROC, and accuracy for most Ansible modules. We deem our approach can contribute to the current research by tackling IaC Defect Prediction from a different perspective and providing a solid baseline for future studies focusing on linguistic issues.

In this paper, we extend our previous work (Borovits et al. 2020) by making the following additional contributions:

1. We compare the performance of six machine Learning algorithms for inconsistency detection: Random Forest, Support Vector Machine, eXtreme Gradient Boosting, Convolutional Neural Networks, Short-Term Memory Networks, and Multi-layer Perceptron.
2. We analyze the impact of different word embedding techniques on the performance of the considered classifiers.
3. We provide a fully comprehensive online appendix^1^ consisting of the FindICI source code, the raw data, and the scripts to replicate our results.

### Structure of the paper

Section 2 describes background. Section 3 details our approach to identify name-body inconsistencies in IaC programs. Section 4 defines the empirical evaluation of the proposed approach, which results are described in Section 5. We discuss the threats to validity in Section 6. Section 7 summarizes the related work in the field and highlights our research contributions. Finally, Section 8 concludes the paper and outlines future works.

## Infrastructure-as-Code and their Linguistic Inconsistencies

This section provides a brief overview of IaC and Ansible, the learning algorithms, and the word embedding models that we used.

### Infrastructure-as-Code and Ansible

Infrastructure-as-Code (IaC) is a model for provisioning and managing computing environments based on the definition of the desired state using source code. IaC relies on software engineering principles, methodologies, and tools. On the one hand, IaC Domain-Specific Languages enable defining the environment state as a software program. On the other hand, IaC tools enable managing the environment based on such programs. In this study, we consider the Ansible IaC language, one of the most popular languages amongst practitioners, according to our previous survey (Guerriero et al. 2019).

In Ansible, a *playbook* defines an IT infrastructure automation workflow as a set of ordered *tasks* over one or more *inventories* consisting of managed infrastructure nodes. A *module* represents a unit of code that a task invokes and serves a specific purpose, such as creating a configuration file from a file template, copying a file, and installing a software package. The definition of a task is essentially a configuration of the module used by the task. A *role* can be used to group a cohesive set of tasks and resources that together accomplish a specific goal, such as installing and configuring JBoss server, and creating a MySQL database instance.

**Fig. 1:**
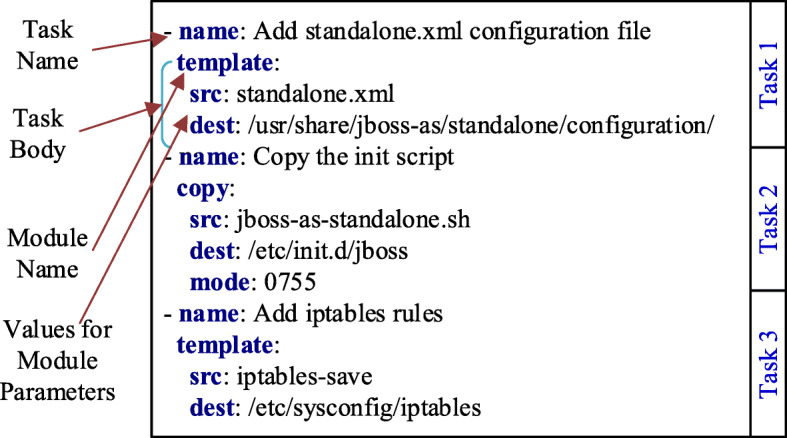
A snippet of an Ansible role, showing three tasks

Figure 1 shows an Ansible snippet for configuring a JBoss server instance and iptables. The first two tasks use the Ansible modules *template* and *copy* to generate the JBoss configuration file from a template, and to copy the JBoss initialization script, respectively. The third task employs the module *template* to create and add firewall rules for the Linux iptables utility. Besides, each module contains parameters (or arguments), for example, *src* and *dest*, that describe the desired state of the system and can be used to manage operations provided by that module.

### Linguistic Inconsistencies in Ansible Tasks

**Fig. 2:**
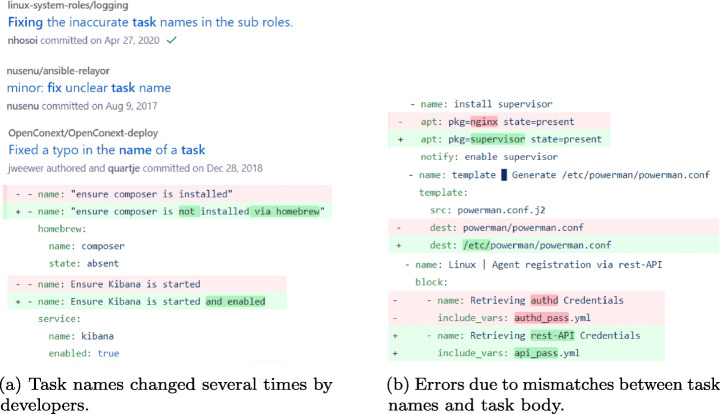
Examples of inconsistent task names and bodies in IaC.

Figure 2 shows some excerpts of commit messages, highlighting inconsistencies and fixes from real-world word Ansible projects collected for our experiments. Although the recommended best practice is to provide a meaningful name to a task,^2^ as shown in Fig. 2a, developers strive to follow this best practice. In the two tasks examples, their names contradict or inaccurately represent what they actually do. For example, if the value of the parameter *state* of the module *homebrew* is “absent”, then, the package composer is uninstalled. Furthermore, as shown in Fig. 2b, the mismatches between task names and task body may be a good indicator of an erroneous task. For example, the first task installs the package *nginx* instead of the package *supervisor*, but the name of the task says that the package should be *supervisor*. Thus, this name-body inconsistency indicates a buggy task.

The aforementioned inconsistencies in task names and bodies can be considered linguistic anti-patterns (Arnaoudova et al. 2013). The presence of linguistic anti-patterns can mislead developers as they can make wrong assumptions about the code behavior or spend unnecessary time and effort to clarify it when understanding source code for their purposes (Arnaoudova et al. 2016). Therefore, highlighting their presence is essential for producing easy-to-understand code. *Our goal is to develop an approach to detect name-body inconsistencies in Ansible tasks.* Although there may exist inconsistencies between code documentation (task or role level comments) and tasks, we could only find a few task examples with comments. Thus, we solely focus on name-body inconsistencies in this study.

In Ansible, the name and body of a task differ from those of a regular method in general programming languages. The task name is a complete sentence or a fragment, and the task body is a configuration of a specific Ansible module. A task only uses a single module, while the tasks using the same module mostly differ in terms of module parameters used and their values. In contrast, the body of a regular method can include arbitrary complex logic, use many APIs (analogous to Ansible modules), and define comments for each line of the code.

## FindICI: A Framework for Learning to Detect Code-Description Inconsistencies in Infrastructure Codes

**Fig. 3:**
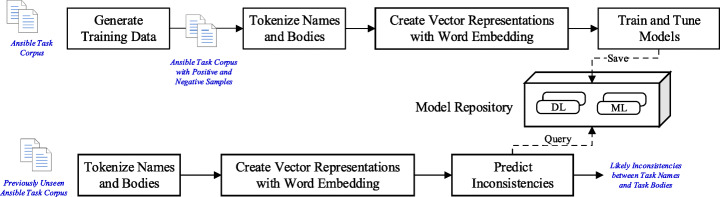
Overview of the FindICI approach

This section presents FindICI, our approach to identifying inconsistencies between natural language descriptions and logic/bodies in IaC code units and, in particular, in Ansible tasks. Figure 3 illustrates the workflow of FindICI as a set of steps, which can be summarized as follows. Finding a sufficient number of real buggy task examples containing inconsistencies is challenging. Therefore, FindICI applies code transformations to generate a corpus of inconsistent Ansible tasks. Both task names and bodies are tokenized and converted into their vector representations that a learning algorithm can use. Afterward, FindICI trains and evaluates binary classifiers using different machine learning and deep learning algorithms and stores them in a model repository. The classifiers can then predict name-body inconsistencies of unseen Ansible tasks based on the module at hand, where the unseen Ansible tasks are tokenized and converted to vectors in the same way as the ones used for training. The following section provides more details about each step.

### Generating Training and Test Data

Our linguistic anti-pattern detection is a binary supervised classification task. Thus, we need a dataset that includes correct (name-body consistent) and potentially buggy (name-body inconsistent) task examples. As Ansible is a relatively new domain-specific language, it is non-trivial to collect a sufficient number of buggy examples from real-world corpus. Inspired by the training data generation in the defect prediction literature (Pradel and Sen 2018; Li et al. 2020), we use simple code transformations to generate the buggy task examples from a given corpus of likely correct task examples by applying simple code transformations. In particular, we swap the body of a given task with another randomly selected task to create inconsistencies. We consider two cases: (i) tasks using the same module (e.g., two tasks with the *template* module) and (ii) tasks using different modules (e.g., one task with the *template* module and another with the *copy* module). Consider the three tasks in Fig. 1: swapping the bodies of Task 1 and Task 3 is an example for the first case; replacing the bodies of Task 1 and Task 3 with the body of Task 2 is an example for the second case.

### Tokenization of Names and Bodies

This step converts the Ansible task descriptions (i.e., task names and task bodies) to a stream of tokens consumed by the learning algorithms. On the one hand, task names are generally short texts in natural language. Therefore, we tokenize them into words. On the other hand, the body of a task has a structured representation. Hence, we use the abstract syntax tree (AST) of the task body to generate the token sequences while preserving the code semantic. In the research literature, ASTs are commonly used for representing code snippets as distributed vectors (Liu et al 2019; Alon et al. 2019). A task body defines an Ansible module’s configuration and instance as a set of parameters (name-value pairs). The tasks can also specify notify actions, conditionals, and loops. The notify actions are to inform other tasks and handlers about the changes to the state of a resource managed by a module. We create an AST model to capture the key information of a task body.

**Fig. 4:**
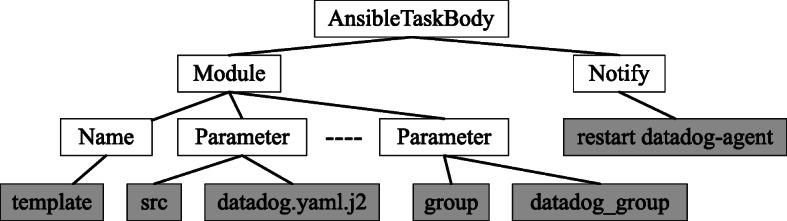
AST model for a task using *template* module

Figure 4 shows a snippet of the generated AST model for the task example in Fig. 1. AST node types capture the semantic information such as modules and their parameters and notify action, and the raw code tokens capture the raw text values. The token stream generated from the AST will be *[AnsibleTaskBody, Module, Name, template, Parameter, src, datadog.yaml.j2, ...., Notify, restart datadog-agent]*.

### Creating Vector Representations

To feed the learning algorithms, the token sequences have to be transformed into vectors. Therefore, we rely on word embedding learning models for generating the vector representation from the Ansible task names and the task bodies. We create a sequence of tokens for each task containing its name and body. We provide this sequence as input of the word embedding model, which takes a set of token string sequences as inputs and produces a map between string tokens and numerical vectors (Mikolov et al. 2013). We generate a corresponding feature vector per word embedding technique for every word in the input task. Word embeddings embed tokens into numerical vectors and place semantically similar words in adjacent locations in the vector space. As a result, the semantic information from the input text is preserved in the corresponding vector representation. Before applying word embeddings, we remove all special characters (e.g., symbols and punctuation) and merge the token sequences for task names and bodies (per task). These steps enable us to build a single vector space for each task as successfully done by previous work on code-comment inconsistency detection for each source code method (Corazza et al. 2018; Pradel and Sen 2018).

### Training and Tuning Prediction Models

We use the learning algorithms to build our binary classifier to categorize the tasks into name-body consistent or not. The embedded token vectors of Ansible tasks generated by the word embedding models are used as input for the classifier. Section 4 provides an overview of the hyperparameter settings used in our experiments. Before feeding the input token vectors into the classifiers, we padded them as appropriate to comply with the fix-width input representations of the classifiers. Motivated by Wang et al. (Wang et al. 2016), we appended zero vectors at the end of the token sequences to reach the size of the longest token sequence of the input tasks. To compute the maximum length of the input sequences *s* we used the equation: *max*_*length*_*s*_ = *mean*_*s*_ + *standard*_*deviation*_*s*_. To avoid long sequences with many padded zeros, we set the max length of the input sequences within two standard deviations of the mean (Moore et al. 2015). This way, we filtered outliers by reducing noise from the padded zeros, and only the 3*%* of the input token sequences were affected by this operation.

### Inconsistency Identification

Inconsistency identification is a binary classification task since the test data are labeled in two classes: *inconsistent* and *consistent*, which are the positive and negative classes in this work. Once the binary classifier is trained with a sufficiently large amount of training data, we can query it to predict whether unseen Ansible tasks (e.g., unseen test data sets) have name-body inconsistencies.

### Implementation

To parse Ansible tasks and build ASTs for them, we developed a custom python tool. We tokenized and lemmatized the task names using the *NLTK library*^3^. We used the *Word2vec*, *Doc2vec*, and *FastText* implementations available in the *gensim* library to generate vectors from tokens. We implemented Machine Learning and Deep Learning models using *TensorFlow*^4^ and *Keras*^5^. We used PyGithub^6^ and PyDriller (Spadini et al. 2018) to locate repositories that contain Ansible IaC scripts. The complete prototype implementation of FindICI, including data set and evaluation results is available on GitHub^7^. It has been integrated into the SODALITE^8^ toolchain that supports guided model-driven engineering of IaC for deploying and managing complex heterogeneous applications (Di Nitto et al. 2020; Kumara et al. 2021).

## Empirical Study Definition and Design

This section describes the design of the empirical study we performed to verify the extent to which FindICI can detect textual inconsistencies in Infrastructure-as-Code. In detail, we aim at understanding whether Machine Learning and Deep Learning classifiers can be used to detect such inconsistencies when trained using word embeddings.

### Research Questions

We set the following research questions:

**RQ1.**: *To what extent can Machine Learning be employed to detect linguistic inconsistencies in IaC?*
**RQ2.**: *To what extent can word embedding representations affect the performance?*
**RQ3.**: *To what extent can the approach find linguistic inconsistencies in real-world IaC scripts?*

In the first research question, we fixed the word embedding representation to *Word2vec using Continuous Bag of Words* (i.e., *Word2vec-CBOW* ) and empirically evaluated and compared six machine-learning models, namely Random Forest (RF), Support Vector Machine (SVM), and eXtreme Gradient Boosting (XGBoost), Multi-Layer Perceptron (MLP), Convolutional Neural Networks (CNNs), and Long-Short Term Memory (LSTM). We selected the Word2Vec-CBOW method as it was one of the two best performing models across classifiers. There are several alternatives to *Word2vec-CBOW*, and we empirically compared these embedding techniques to check their impact on the performance of the classifiers in **RQ2**.

All experiments were performed on a machine with an Intel Core i7-9750H CPU, 16GB of memory, and a single NVIDIA Quadro P2000 GPU.

### Data Collection

To answer **RQ1** and **RQ2**, we evaluated FindICI on a real-world corpus of Ansible tasks mined from GitHub. To ensure the quality of the data collected, we used the following criteria adapted from Rahman and Williams (2018) and Dalla Palma et al. (2021).

**Criterion 1**: - At least 11% of the files belonging to the repository must be IaC scripts.
**Criterion 2**: - The repository has at least 10 contributors.
**Criterion 3**: - The repository must have at least two commits per month.
**Criterion 4**: - The repository is not a fork.

These criteria were used by previous works to collect IaC scripts (Dalla Palma et al. 2021; Rahman and Williams 2018). In particular, criterion 1 represents a cut-off to ensure that only repositories with a sufficient amount of IaC scripts and commit history are analyzed. Indeed, Jiang and Adams (2015) observed that in open-source repositories a median of 11% of the files are IaC scripts.

We found 38 GitHub repositories that met the above criteria. We extracted 18,286 Ansible tasks from them. As we trained the corresponding ML and DL models for each Ansible module, our experiments only considered the 10 most used modules, which account for 10,396 tasks in the collected data set. Table 1 shows the distribution of the data samples for each module type in the collected dataset. We applied the transformations described in Section 3.1 to create our dataset. Thus, the resulting dataset comprises 20,792 observations with a balanced number of instances for each label.

**Table 1:** Size of the collected instances per each module

Module	shell	command	set_fact	template	file	copy	gather_facts	service	debug	fail
**Tasks**	2,126	1,702	1,246	1,198	1,151	773	752	569	484	395

To answer **RQ3**, we focused on the data collected by Dalla Palma et al. (2021) for defect prediction of Ansible code. The dataset provides over 180*k* observations of defect-prone and defect-free IaC blueprints collected from 85 open-source GitHub repositories based on the Ansible language. From the Ansible files present in the dataset, we extracted 14,116 tasks that we use to validate the best performing model. In addition, we ensured that there is not any information leakage between this dataset and ours by filtering out any common tasks. Table 2 shows statistics about the most recurring modules.

**Table 2:** Size of the collected instances per module for the external dataset

Module	file	shell	set_fact	command	template	copy	service	fail	debug	gather_facts
**Tasks**	2,617	2,297	2,236	2,200	2,025	1,111	791	503	245	91

### Classifiers Selection

To address the first research question, we relied on six classification algorithms, namely *Random Forest* (RF) (Ho 1995), *Support Vector Machine* (SVM) (Cortes and Vapnik 1995), *eXtreme Gradient Boosting* (XGBoost) (Chen and Guestrin 2016), *Multi-Layer Perceptron* (MLP) (Haykin 1998), *Convolutional Neural Networks* (CNNs) (Matsugu et al. 2003), and *Long-Short Term Memory* (LSTM) (Cheng et al. 2016), as they have been widely used for text classification and defect prediction (Fakhoury et al. 2018; Li et al. 2020; Liu et al 2019; Minaee et al. 2021; Omri and Sinz 2020; Pradel and Sen 2018).

More specifically, we selected RF for its robustness to noise and correlated variables and low proneness to overfitting (Ho 1995). Likewise, SVM was selected for its low proneness to overfitting and its ability to handle non-linear data (Cortes and Vapnik 1995). On the other hand, XGBoost allows for loss function customization and it is less biased by unbalanced datasets (Chen and Guestrin 2016). Concerning the neural network based algorithms, we selected MLP as a baseline neural network for its simplicity (Haykin 1998), and CNNs and LSTM to verify whether their more complex nature provides better performance for detecting inconsistency (Cheng et al. 2016; Matsugu et al. 2003).

### Model Selection

The model selection was guided by a *grid search* on the models’ parameters^9^ through a *stratified k-folds* cross-validation. Grid search is an exhaustive search algorithm through a manually-specified subset of parameters, while stratified k-folds cross-validation is a widely used validation method that ensures that every observation from the dataset has the chance of appearing in the training and test set (James et al. 2013). It randomly partitions the data into ten folds of equal size, applying a stratified sampling (e.g., each fold has the same proportion of inconsistencies). A single fold is used as the test set, while the remaining ones are used as the training set. The process was repeated ten times, using each time a different fold as the test set. Then, the model performance was reported using the mean achieved over the ten runs. Please consider that we could not employ this strategy for CNNs and LSTM as it was too computationally expensive. Therefore, we manually calibrated the classifiers and we applied *hold-out validation* (James et al. 2013). We split the dataset into three sets (i.e., 60% training, 20% validation, and 20% test) with the same distribution of inconsistencies.

### Model Validation

The built models are used to predict task-body inconsistencies. As usual in machine learning, there are four possible prediction outcomes:

- True Positive (TP): when the actual class is inconsistent and the predicted class is also inconsistent.
- False Negative (FN): when the actual class is inconsistent but the predicted class is consistent.
- True Negative (TN): when the actual class is consistent and the predicted class is also consistent.
- False Positive (FP): when the actual class is consistent but the predicted class is inconsistent.

To evaluate the performance of the trained models, we used the common metrics used in binary classification problems, namely *accuracy*, *precision*, *recall*, *F1 score*, *MCC* (Matthews Correlation Coefficient), and *AUC-ROC* (Area Under the Receiver Operating Characteristic curve).

$$Accuracy = \frac{TP+TN}{TP+TN+FP+FN}$$

$$Precision = \frac{TP}{TP+FP}$$

$$Recall = \frac{TP}{TP+FN}$$

$$F1-score = \frac{precision \times recall}{precision + recall}$$

$$MCC = \frac{TP \times TN - FP \times FN}{\sqrt{(TP + FP)(TP + FN)(TN + FP)(TN + FN)}}$$

AUC measures the entire two-dimensional area underneath the entire ROC (receiver operating characteristic curve), which plots true positive rate and false positive rate. A good classifier has an AUC closer to 1, whereas, a poor model has an AUC near to 0. Please consider that we used AUC to tune the models when applying cross-validation. To analyze the classifiers’ performance we reported the following evaluation measures: the performance is analyzed in terms of mean and standard deviation.

Afterwards, to compare performance across classifiers and word embedding techniques, we followed the recommendations in Demšar (2006). In particular, first, we applied the Friedman test (Friedman 1940) with a significance level equal to 0.05 to reject the null hypothesis. Once we have established a statistical difference between the classifiers’ performance, we applied the pairwise posthoc analysis recommended by Benavoli et al. (Benavoli et al. 2016), where the average rank comparison is replaced by a Wilcoxon signed-rank test (Wilcoxon 1992) with Holm’s alpha correction (Holm 1979). To statistically compare the performance of multiple classifiers and multiple word embedding methods over multiple Ansible modules, we plotted the results using several critical difference (CD) diagrams (Demšar 2006), which visualize the results of the Wilcoxon-Holm post hoc test. In a CD diagram, the positions of the treatments (e.g., classification or word embedding methods) represent their average ranks across all outcomes of the observations. Two or more treatments are connected with each other with a thick horizontal line if they are not significantly different in terms of the considered metric. To perform this statistical analysis and draw CD diagrams, we relied on the implementation provided by Ismail Fawaz et al. (2019).

We also perform a qualitative analysis of classification outcomes. We employ the t-Distributed stochastic neighbor embedding (t-SNE) (Van der Maaten and Hinton 2008) to visualize in the dimension space the words of a predicted true positive task and a false positive task. The t-SNE is a dimensionality reduction technique that has been widely used in the Natural Language Processing (NLP) literature to project the relationship between words in a corpus in the two-dimensional space (Wattenberg et al. 2016; Van Der Maaten 2014; Gisbrecht et al. 2015). In our study, we created a distributed vector representation of size 100 for each word using the corresponding word embedding techniques. Thus, we used t-SNE to reduce the dimensionality of the vectors and project the learned relationships between the words in the two-dimensional space. The words used for consistent tasks should be placed relatively closer in the feature space than the corresponding words of the inconsistent task. Particularly, we expect the words forming the task name to be placed close to the words of the task body of the consistent task. On the other hand, we expect the words that compose the task name to be placed relatively further from those that constitute the task body for the inconsistent task.

### Word Embedding Selection

To answer **RQ2**, we chose three widely used word embedding learning models, *Word2vec* (Mikolov et al. 2013), *Doc2vec* (Le and Mikolov 2014), and *FastText* (Joulin et al. 2016). These embedding models are used by software engineering research for learning representations source codes and method names (Pradel and Sen 2018; Liu et al 2019; Li et al. 2020; Fakhoury et al. 2018), and other natural language texts (Sulistya et al. 2020). *Word2vec* is a two-layer neural network that processes text by creating vector representations from words. Word2vec can use either continuous bag-of-words (CBOW) or continuous skip-gram to learn a distributed representation of the words. CBOW enables predicting a single word from a fixed window size of context words (or surrounding words), whereas Skip-gram predicts several context words from a single input word. *Doc2Vec* learns fixed-length feature representations from variable-length pieces of texts, such as sentences, paragraphs, and documents. It extends *Word2vec* by considering the ordering and semantics of the words within blocks of texts. *Doc2vec* can use two model architectures: Distributed Bag of Words of Paragraph Vector (PV-DBOW) and Distributed Memory of Paragraph Vector (PV-DM), which are analogous to Skip-gram and CBOW implemented by *Word2vec*. Doc2vec generates a single vector representation for every word among all documents in the corpus by considering the additional context of the document. In addition to this vector, it generates a vector per document. However, to maintain the compatibility of Doc2vec with the rest of our word embedding models, we did not use such a document-level vector. Finally, *FastText* improves on *Word2vec* by taking word parts (e.g., prefixes, roots, and suffixes) into account, enabling the embedding training on smaller datasets and generalizing to unknown words.

In a recent study, Sulistya et al. (2020) compared different word embedding learning methods for finding software-relevant tweets. Following their guidelines, we used the same hyper-parameter settings for each word embedding learning model (i.e., *Word2vec*, *Doc2Vec*, and *FastText*). We choose the following key parameters: context window size (6) and vector size (100). The context window defines the number of words that are used to determine the context of each word. As the Ansible tasks are short texts, we use a window size of 6. The vector size is the dimensionality of vector embeddings to be learned. According to the previous studies (Pennington et al. 2014; Mikolov et al. 2013), 100-400 is the most frequently used setting, and the best accuracy is achieved with 300 tokens. However, since the corpus and the vocabulary (the number of unique words) is small, we choose 100 tokens, which is also the default value used by our implementation (i.e., *gensim*), to prevent overfitting.

## Results of the Empirical Study

This section reports the results of the empirical study previously defined.

### RQ1. To what extent can Machine Learning be employed to detect linguistic inconsistencies in IaC?

**Fig. 5:**
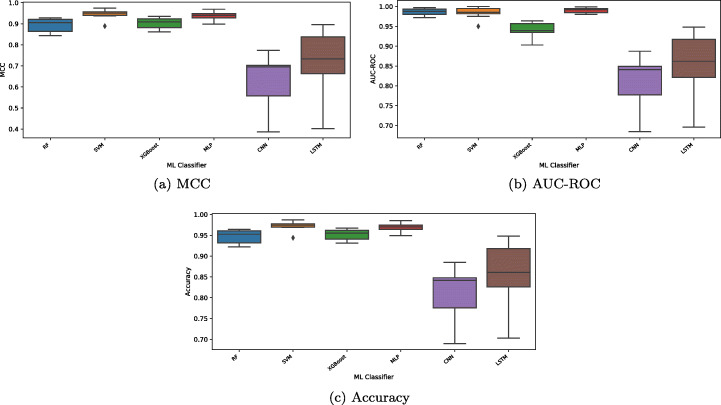
Boxplots depicting MCC, AUC-ROC, and accuracy for each classifier

Tables 3, 4, 5, 6, 7 and 8 summarize the performance of the selected classifiers to detect linguistic inconsistency on the 10 most used modules in Ansible. Figure 5 depicts the boxplots for the MCC, AUC-ROC, and accuracy metrics.

**Table 3:** Results for all considered metrics achieved on the 10 most used modules in Ansible using Support Vector Machine

	shell	command	set_fact	template	file	gather_facts	copy	service	debug	fail
**AUC**	0.99	0.97	1.00	0.99	0.99	0.99	0.99	1.00	1.00	1.00
**MCC**	0.94	0.89	0.95	0.95	0.96	0.95	0.97	0.94	0.94	0.96
**Accuracy**	0.97	0.94	0.98	0.98	0.98	0.97	0.99	0.97	0.97	0.98
**F1 score**	0.97	0.95	0.97	0.98	0.98	0.97	0.99	0.97	0.97	0.98
**Precision**	0.96	0.93	0.98	0.96	0.97	0.95	0.99	0.96	0.96	0.97
**Recall**	0.98	0.97	0.97	0.99	0.99	1.00	0.99	0.98	0.98	0.99

**Table 4:** Results for all considered metrics achieved on the 10 most used modules in Ansible using Random Forest

	shell	command	set_fact	template	file	gather_facts	copy	service	debug	fail
**AUC**	0.98	0.97	0.99	0.98	0.99	0.99	0.98	0.99	0.99	0.99
**MCC**	0.87	0.84	0.86	0.89	0.92	0.92	0.84	0.92	0.93	0.92
**Accuracy**	0.94	0.92	0.93	0.95	0.96	0.96	0.92	0.96	0.96	0.96
**F1 score**	0.94	0.93	0.93	0.95	0.96	0.96	0.92	0.96	0.96	0.96
**Precision**	0.94	0.92	0.91	0.92	0.95	0.94	0.92	0.98	0.96	0.95
**Recall**	0.93	0.93	0.95	0.98	0.97	0.98	0.92	0.95	0.97	0.97

**Table 5:** Results for all considered metrics achieved on the 10 most used modules in Ansible using eXtreme Gradient Boost

	shell	command	set_fact	template	file	gather_facts	copy	service	debug	fail
**AUC**	0.95	0.93	0.96	0.94	0.97	0.98	0.93	0.96	0.95	0.96
**MCC**	0.90	0.86	0.93	0.88	0.93	0.93	0.86	0.91	0.91	0.91
**Accuracy**	0.95	0.93	0.96	0.94	0.97	0.97	0.93	0.96	0.95	0.96
**F1 score**	0.95	0.94	0.96	0.94	0.97	0.97	0.93	0.96	0.95	0.95
**Precision**	0.95	0.92	0.95	0.93	0.96	0.96	0.93	0.97	0.96	0.95
**Recall**	0.95	0.95	0.97	0.97	0.98	0.97	0.93	0.95	0.95	0.96

**Table 6:** Results for all considered metrics achieved on the 10 most used modules in Ansible using Multi-Layer Perceptron

	shell	command	set_fact	template	file	gather_facts	copy	service	debug	fail
**AUC**	0.98	0.98	0.99	0.99	0.99	0.98	0.99	1.00	1.00	0.99
**MCC**	0.91	0.90	0.94	0.92	0.94	0.95	0.94	0.96	0.97	0.95
**Accuracy**	0.96	0.95	0.97	0.96	0.97	0.97	0.97	0.98	0.98	0.97
**F1 score**	0.96	0.95	0.97	0.96	0.97	0.97	0.97	0.98	0.98	0.97
**Precision**	0.95	0.93	0.96	0.95	0.96	0.96	0.95	0.98	0.99	0.97
**Recall**	0.96	0.98	0.97	0.98	0.98	0.99	0.99	0.98	0.98	0.97

**Table 7:** Results for all considered metrics achieved on the 10 most used modules in Ansible using Long-Short Term Memory

	shell	command	set_fact	template	file	gather_facts	copy	service	debug	fail
**AUC**	0.86	0.93	0.81	0.939	0.948	0.84	0.89	0.87	0.76	0.70
**MCC**	0.73	0.85	0.65	0.88	0.90	0.70	0.79	0.74	0.55	0.40
**Accuracy**	0.85	0.93	0.82	0.94	0.95	0.84	0.89	0.87	0.76	0.70
**F1 score**	0.84	0.93	0.85	0.93	0.95	0.83	0.87	0.86	0.78	0.74
**Precision**	0.95	0.94	0.78	0.90	0.95	0.93	0.98	0.90	0.69	0.69
**Recall**	0.75	0.92	0.93	0.97	0.95	0.75	0.79	0.82	0.90	0.80

**Table 8:** Results for all considered metrics achieved on the 10 most used modules in Ansible using Convolutional Neural Networks

	shell	command	set_fact	template	file	gather_facts	copy	service	debug	fail
**AUC**	0.85	0.76	0.77	0.84	0.89	0.80	0.84	0.85	0.84	0.68
**MCC**	0.70	0.52	0.54	0.69	0.77	0.61	0.70	0.71	0.70	0.39
**Accuracy**	0.85	0.76	0.76	0.84	0.88	0.80	0.85	0.85	0.84	0.69
**F1 score**	0.84	0.76	0.77	0.86	0.88	0.82	0.87	0.86	0.85	0.73
**Precision**	0.90	0.72	0.72	0.80	0.93	0.81	0.82	0.79	0.77	0.66
**Recall**	0.79	0.80	0.84	0.92	0.84	0.84	0.93	0.94	0.95	0.83

Overall, we can observe that all ML models perform similarly with the best results achieved by SVM. This classifier yields an accuracy ranging from 0.94 to 0.99, MCC from 0.89 to 0.97, and AUC from 0.97 to 1. It detects inconsistent tasks with F1 score ranging from 0.95 to 0.99, recall from 0.97 to 1, precision from 0.93 to 0.99. Among the neural-network based classifiers, the MLP classifier performed the better in terms of all evaluation metrics and the CNN model is the worst performer. MLP yielded an accuracy ranging from accuracy from 0.95 to 0.99, MCC from 0.90 to 0.97, and AUC from 0.98 to 1.

On the other hand, it finds inconsistent tasks with F1 score ranging from 0.95 to 0.98, recall from 0.96 to 0.99, precision from 0.93 to 0.99.

**Fig. 6:**
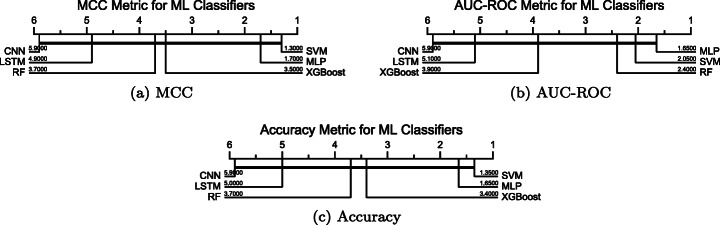
Critical Difference diagram based on the Wilcoxon-Holm test to detect pairwise significance between the performance achieved by the considered classifiers: MCC, AUC-ROC, and accuracy metrics

Figure 6 depicts the result of the statistical analysis we conducted on all the considered ML classifiers. Looking at Support Vector Machine, eXtreme Gradient Boosting, and Random Forest, we can notice that although the former is the best-performing classifier over eight Ansible modules in terms of the metrics MCC, AUC-ROC, and accuracy. For both accuracy and MCC metrics, there is no difference between RF and XGBoost models. Furthermore, considering the AUC-ROC metric, SVM and RF perform similarly. The results also show that the differences between the performance of the three neural networks-based classifiers are statistically significant (there is no a think line connecting the classifiers). Although in a different context (extracting entities from textual medical records using word embeddings and neural networks), our results are similar to those previously shown by Dudchenko and Kopanitsa (2019).

**Fig. 7:**
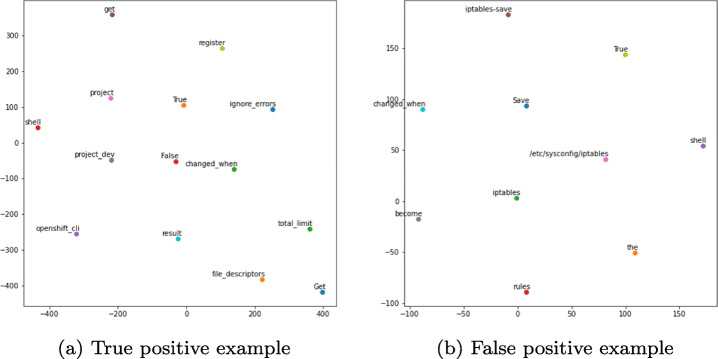
The t-SNE visualizations of the words of a true positive predicted observation and a false positive predicted observation of the SVM ML model

**Fig. 8:**
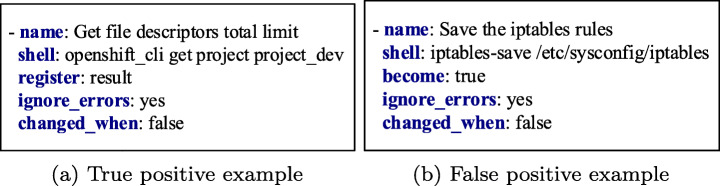
Ansible tasks used in the t-SNE visualizations of Fig. 7

Figure 7 shows the t-SNE results for the classification results depicted in Fig. 8. We used the SVM classifier, which is the best-performing model. We observed that the words of the task name are positioned relatively closer to the words of the task body for the predicted false positive task compared to the corresponding word positioning for the predicted true positive task. For example, the words *Save*, *iptables* are relatively closer to the words *iptables-save*, */etc/sysconfig/iptables*, and *become* in the false-positive example. In contrast, the words *Get*, *file_descriptors*, *total_limit* are positioned relatively far from the words *openshift_cli*, *get*, and *project* in the true positive example. Please note that the scales are different in the two figures. However, all words of task names and bodies are relatively closer in case of false positives than true positives.

The explanation for the erroneous classification for the false positive observation lies within the collected Ansible tasks. Most of the misclassified tasks contain words of low occurrence frequency in the rest of the tasks. Thus, these observations are too few for the classifier to learn to predict them accurately. For example, for the demonstrated false-positive task illustrated in Figs. 7 and 8, the combination of the words *Save* and *iptables* does not exist in any other task in the dataset. Therefore, such observations are treated as outliers that lead to wrong predictions. This pattern is observed for most of the misclassified observations. Finally, the rest of the misclassifications occur because some words in the tasks of the test set do not exist in the corpus of words of the word embedding model used during the training phase. As a result, the classifiers miss feature representations of some words and erroneously classify the tasks. To reduce these misclassification errors in the future, we will need a bigger Ansible tasks corpus to train the ML models to perform the classification task accurately.

**Fig. 9:**
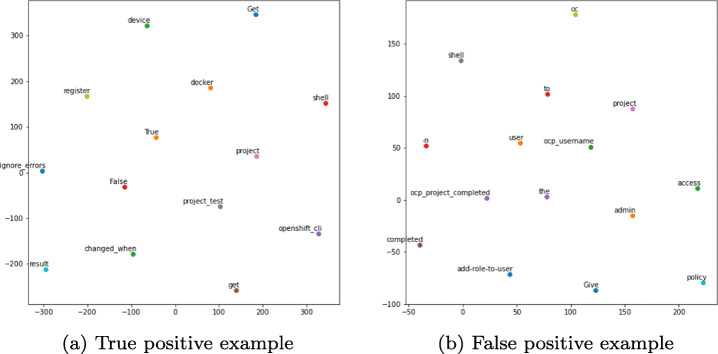
The t-SNE visualizations of the words of a true positive predicted observation and a false positive predicted observation for the CNN deep learning model

**Fig. 10:**
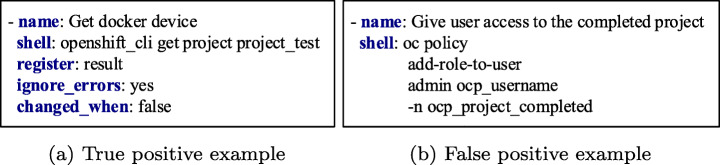
Ansible tasks used in the t-SNE visualizations of Fig. 9

We also qualitatively analyzed the classification results of a deep learning model, namely the CNN model. Analyzing the visualizations in Fig. 9 for the classified tasks in Fig. 10, we can observe the same pattern for the word positioning between the predicted true positive example and the false positive example as remarked above. Namely, the words contained in the false-positive example are placed closer in the dimension space than the corresponding words of the true positive example. This observation indicates that the task was indeed falsely classified as inconsistent. The reasoning for the occurring misclassifications is the same as above, leading to the lower performance of the DL models compared to the corresponding performance of the ML models. DL models require a large text corpus to make high-quality predictions (Roberts 2016). Consequently, tasks with no vector representations or word combinations that do not occur in other tasks will ultimately result in erroneous predictions. 
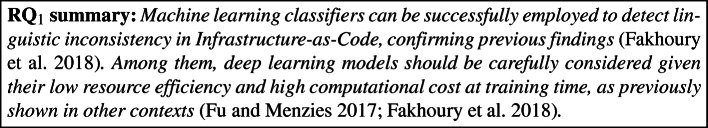

### RQ2. To What Extent can Word Embedding Representations Affect the Performance?

**Fig. 11:**
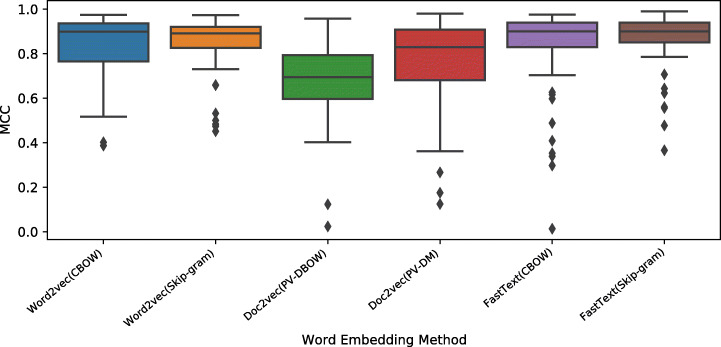
Boxplots representing the MCC values obtained by the word embedding methods for Ansible name-body inconsistency detection

**Fig. 12:**
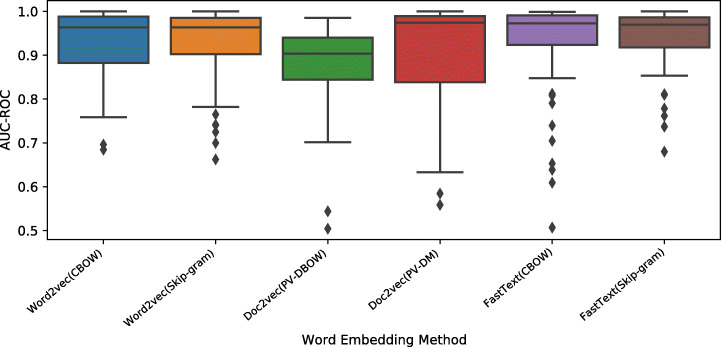
Boxplots representing the AUC-ROC values obtained by the word embedding methods for Ansible name-body inconsistency detection

**Fig. 13:**
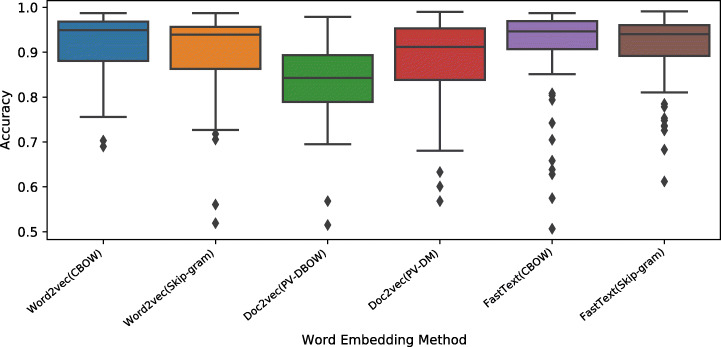
Boxplots representing the accuracy values obtained by the word embedding methods for Ansible name-body inconsistency detection

Figures 11, 12 and 13 show the boxplots for the MCC, AUC-ROC, and accuracy values obtained by applying different word embedding techniques with the six classifiers over eight Ansible modules. The online appendix includes the detailed experimental results of the six classifiers for each embedding method. Generally, all models have high performance in terms of the considered metrics. However, the models based on *Word2vec* and *FastText* achieve the best results, and their variants based on Skip-gram have a smaller performance variance. Looking at *Doc2vec*, the model based on *PV-DM*, which is analogous to *Word2vec CBOW*, performs better than the ones based on *PV-DBOW* which is analogous to Word2vec Skip-gram.

**Fig. 14:**
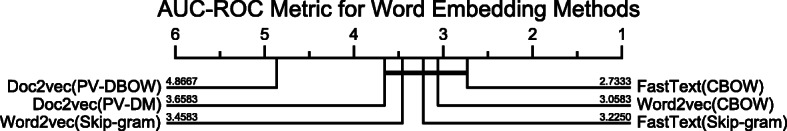
Critical Difference diagram based on the Wilcoxon-Holm test to detect pairwise significance between the AUC-ROC achieved by the considered techniques for word embedding

**Fig. 15:**
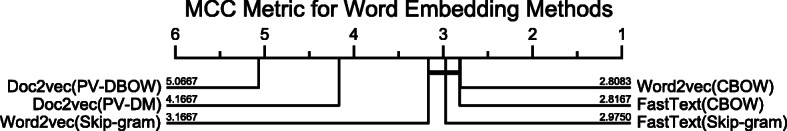
Critical Difference diagram based on the Wilcoxon-Holm test to detect pairwise significance between the MCC achieved by the considered techniques for word embedding

**Fig. 16:**
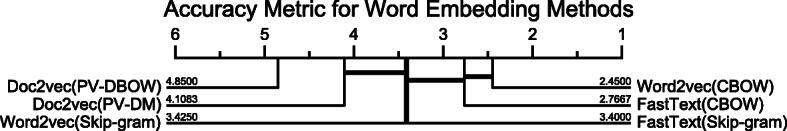
Critical Difference diagram based on the Wilcoxon-Holm test to detect pairwise significance between the accuracy achieved by the considered techniques for word embedding

The results are confirmed by the statistical analysis, which results are depicted in Figs. 14, 15 and 16. Generally, the word embedding models *Word2vec* and *FastText* achieve the best results in terms of the considered metrics, and the *Doc2vec* model is the worst performer. All the embedding models except the *Doc2vec PV-DBOW* model perform similarly over eight modules in terms of the AUC-ROC and accuracy metrics. There are no statistically significant differences among *FastText* and *Word2vec* models in terms of MCC. Our results confirm previous work (Sulistya et al. 2020; Mikolov et al. 2013) which assessed the superiority of *Word2vec* and *FastText* in a different context (i.e., text mining). In addition, our work agrees with the findings of previous work (Lau and Baldwin 2016) suggesting that *Doc2vec* creates document embeddings which align with lower frequency words when the documents are short and the corpus is relatively small. The maximum index of our corpus consists of 9,651 unique words and the average size of our task sequences is 22 token sequences. Both numbers are relatively low compared to the corresponding numbers of the benchmark NLP task used for the evaluation of the *Doc2vec* model (Le and Mikolov 2014). 
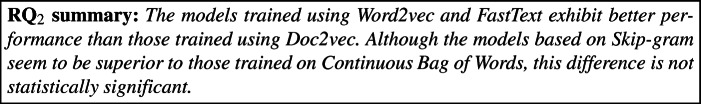

### RQ3. To What Extent can the Approach Find Linguistic Inconsistencies in Real-World IaC Scripts?

To evaluate the effectiveness of our IaC inconsistency detectors, we applied them to unmodified real-world Ansible tasks and manually inspected the reported inconsistencies to assess their precision. We used the best detector, which is the SVM model with Word2vec.

#### Results

To evaluate the best-performing model on a real-world dataset, the first three authors of this paper manually assessed whether the predicted label for a task is correct or not. We addressed all the discrepancies through discussions. Cohen’s Kappa coefficient was 0.786, indicating a substantial agreement. Since the number of tasks in the real-world dataset was relatively high (i.e., 14,116), we examined only a statistically significant sample of 380 tasks selected from the dataset by considering a 95% confidence level and a 5% margin of error. All tasks in the real-world sample had an inconsistent predicted label. This way, we could evaluate the performance of our model based on the number of the predicted false positives. The results suggest that our model correctly detected inconsistency for 193 tasks while falsely predicting 187 tasks. These results are comparable to the results reported in a previous study (Pradel and Sen 2018), which motivated our work regarding the argument swapping transformation for the creation of the inconsistent set.

#### Qualitative Analysis of Inconsistencies and False Positives

Our best-performing model contains vector representations for 9,651 words, which comprise the Ansible tasks during the training phase. The statistically significant sample of the real-world dataset consisted of 4791 unique words. The relevantly average performance of our model can be explained by the fact that only 1,316 common words existed in the pre-trained corpus of our model. In other words, our model knew the features (vector presentations) for only 27% of the words of the real-world dataset. We deem this amount insufficient to predict effectively whether a task is consistent or not since our model did not contain the corresponding vector representations for most of the words in the real-world dataset.

**Fig. 17:**
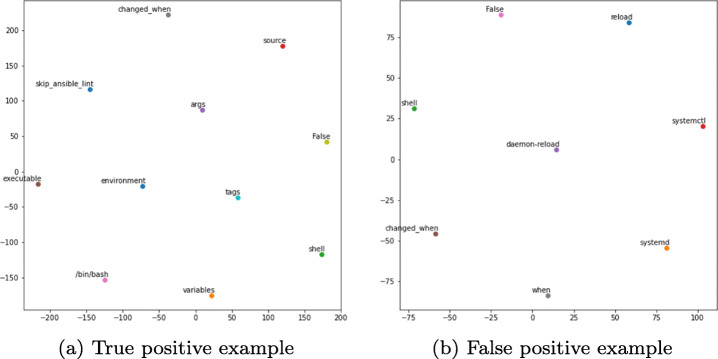
The t-SNE visualizations of the words of a true positive predicted observation and a false positive predicted observation for the real-world dataset

**Fig. 18:**
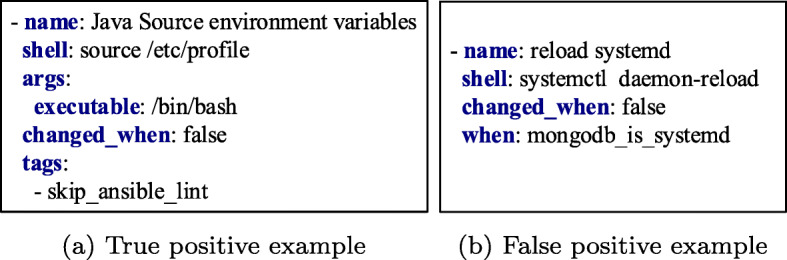
Ansible tasks used in the t-SNE visualizations of Fig. 17


We qualitatively assessed the result for a predicted true positive task and a predicted false positive task by analyzing Fig. 17 and the corresponding tasks on Fig. 18 that confirm the findings reported in the two previous sections. Particularly, we observed that the distances between the words of the false-positive task are closer than those of the true-positive task. For example, regarding the false positive task, the task name words such as *reload* and *systemd* are positioned closer to the corresponding task body words such as *daemon-reload*, *systemctl* and *when*. This result implies that the pre-trained embedding model based on our dataset could successfully detect the relationships between the words in the unseen real-world set even with a relatively small number of common words. However, our classifier is unable to predict the correct labels. Therefore, this confirms our findings from the previous sections, which suggest that the classifiers lack performance when there are missing word representations in the feature space. Finally, given the small number of common words between the tasks of our dataset and the real-world dataset, we note that the sets contain a significant number of unique tasks. This result suggests that to improve the performance of our model, we need to collect and include a higher number of tasks in the training phase.

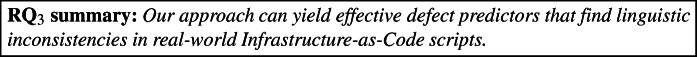


## Threats to Validity

We present the potential threats to the internal, external, and construct validity of our findings.

### Threats to Construct Validity

The collected repositories may not be relevant for the problem at hand. We mitigated this threat by applying the criteria used in previous works on IaC smell detection to ensure the quality of the collected data set. Another threat to construct validity concerns the mutation of scripts employed to generate inconsistent cases, which may not represent real-world inconsistent tasks. Please consider that we created datasets in which consistent and inconsistent programs are equally represented. However, this assumption could not hold, leading to a different class distribution compared to real settings. Nevertheless, we tried to mitigate this threat by applying the existing approaches that have successfully used mutation to generate the training data (Li et al. 2020; Pradel and Sen 2018). We plan to further mitigate this threat by gathering more real-cases of inconsistent Ansible tasks. As discussed in Section 4.6, we leveraged the configurations employed by previous studies for word embedding models. While the selected parameters performed well, experiments with different configurations would have provided some insights into the effectiveness of the word embedding methods.

### Threats to Internal Validity

The choice of the features used to train the classifiers could influence linguistic anti-patterns detection. We mitigated this threat by training the model using several features (obtained by transforming each task to a vector space of words) extracted from more than ten thousand Ansible tasks. The feature engineering for the classification task depends on the quality of the code base, including naming conventions, typos, and abbreviations. This aspect poses a threat to validity, and advanced NLP techniques can be employed to overcome this.

### Threats to External Validity

The conclusions are derived only from a subset of modules in Ansible (i.e., the ten most used), which might not be reproducible for other modules and languages. However, we used both generic modules (such as *command* modules) and more specific modules. Specific modules (e.g., the *copy* module) do focus works, but general modules can execute ad-hoc OS commands. We believe that using a mix of generic and specific modules may mitigate, at least partially, this threat. Finally, we analyzed only Ansible projects, and the results could not generalize to other IaC languages (e.g., Chef, Puppet). Extending our approach to these languages is part of our agenda. We validated our approach with a real-world dataset manually validated by the first three authors. We addressed all the discrepancies through discussions and achieved a percentage of agreement of 89%, with Cohen’s kappa equal to 0.786, which indicates a substantial agreement. Nevertheless, manual analyses present intrinsic bias that could have affected the generalizability of the results.

## Related Work

In this section, we first discuss the existing studies on IaC, which we ground on a recent mapping study on IaC research (Rahman et al. 2019). Then, we overview the linguistic anti-patterns literature for other programming languages.

### Empirical Studies related to IaC

According to the mapping study, IaC has been used to support the automated provisioning and deployment of applications on different infrastructures and implement DevOps and continuous deployment. Several empirical studies focus on testing and quality assurance and the evolution of IaC artifacts to analyze how practitioners adopt this technology. IaC has been used to support the automated provisioning and deployment of applications on different infrastructures and implement DevOps and continuous deployment. Guerriero et al. (2019) identified further insights on the challenges related to the IaC development and testing in industrial contexts by surveying 44 practitioners. Sandobalín et al. (2020) focused on the effectiveness of IaC tools, while Rahman et al. (Islam Shamim et al. 2020; Hasan et al. 2020) on testing and security practices mined from grey literature. With similar goals, the latter analyzed the development practices that contributed to defective IaC scripts (Rahman et al. 2020) and replicated previous studies (Rahman et al. 2021). Finally, Opdebeeck et al. (2020) analyzed the adoption of semantic versioning in Ansible roles, while Kokuryo et al. (2020) examined the usage of imperative modules in the same language.

### IaC Quality and Defect Prediction

Most of the previous works describe infrastructure code quality in terms of smelliness (Folwer 1999) and defects-proneness of Chef and Puppet infrastructure components. From a smelliness perspective, Schwarz et al. (2018), Spinellis et al. (Sharma et al. 2016), and Rahman et al. (2019) applied the well-know concept to IaC, and identified code smells that can be grouped into four groups: (i) *Implementation Configuration* such as complex expressions and deprecated statements; (ii) *Design Configuration* such as broken hierarchies and duplicate blocks; (iii) *Security Smells* such as admin by default and hard-coded secrets; (iv) *General Smells* such as long resources and too many attributes. From a defect prediction perspective, Rahman and Williams (2019b) identified ten source code measures that significantly correlate with defective infrastructure as code scripts such as properties to execute bash and/or batch commands, to manage file permissions, and more. Dalla Palma et al. (2020a, 2020b, 2021) proposed a set of tools to calculate quality metrics for Ansible scripts and projects and use them for predicting defective scripts. Kumara et al (2020) proposed a tool to detect smells in TOSCA scripts using an ontology-based approach. Cito et al. (Schermann et al. 2018) detected violations of Docker best practices, while Dai et al. (2020) leveraged static code analysis and rule-based reasoning to detect risky IaC artifacts. Finally, Sotiropoulos et al. (2020) crafted a tool to identify missing dependencies and notifiers in Puppet manifests by analyzing system call traces.

In this work, we step up this research line by proposing a novel automated approach that employs word embeddings and learning techniques to detect linguistic anti-patterns, focusing on short-text-name-body inconsistencies in IaC code units, in particular Ansible. We focused on Ansible, rather than Puppet and Chef, because Ansible is the most used IaC in the industry (Guerriero et al. 2019). We evaluated the effectiveness of our approach with various machine learning models, deep learning models, and word embedding models.

### Linguistic Anti-patterns Literature in Other Domains

Arnaoudova et al. (2013) coined the term “software linguistic anti-patterns” for the bad practices about naming and documentation in source code. The authors proposed a catalog of such anti-patterns for object-oriented programs and assessed the relevance and usefulness of the catalog with an empirical study with developers (Arnaoudova et al. 2016). They also studied how linguistic anti-patterns can exacerbate design smells and consequently increase the change and fault proneness of source code (Guerrouj et al. 2017). A user study by Fakhoury et al. (2020) showed the negative impact on the cognitive load experienced by developers when reviewing code containing linguistic anti-patterns. The authors also developed anti-pattern detectors using deep neural networks and traditional machine learning (Fakhoury et al. 2018). The evaluation of the detectors with a dataset of Java programs showed that machine learning could outperform deep neural networks. With a large scale dataset of libraries (APIs), Java projects using the APIs, and StackOverflow questions concerning the APIs, Aghajani et al. (2018) studied the impacts of linguistic inconsistencies with the libraries on the chance of introducing bugs in the projects using those libraries. They found a 29% increase in the likelihood of introducing bugs. Palma et al. (2017) proposed a catalog of linguistic anti-patterns in RESTful APIs, which mainly consider bad practices in designing and documenting RESTful APIs. Their anti-pattern detection tool applies semantic similarity checking techniques to detect the inconsistencies between API documentation and API URLs. We believe that our study is the first work that studies the linguistic anti-patterns in IaC programs.

## Conclusion and Future Work

In this paper, we study to what extent machine learning can detect linguistic inconsistencies in Infrastructure-as-Code (IaC). In particular, we propose FindICI, a method to detects linguistic inconsistencies between names and bodies of IaC code units by leveraging word embedding and learning models for classification tasks.

To evaluate our method, first, we generate a synthetic dataset of inconsistencies by applying simple code transformations to create inconsistent tasks from likely consistent tasks. Next, we generate the word embeddings from the tokenized names and bodies of consistent and inconsistent tasks. We used word embedding to train the various binary classifiers for inconsistency detection. We evaluated the effectiveness of our approach with an Ansible dataset composed of 38 open source repositories using six machine learning algorithms (three of which are based on neural networks) and six word embedding models.

Our results confirm that both classical learning algorithms and novel deep learning algorithms with various word embedding methods can be successfully applied to detect linguistic inconsistencies in IaC scripts.

As part of our future agenda, we plan to extend FindICI to detect additional linguistic inconsistencies and misconfigurations in Ansible code scripts. We also aim to extend FindICI to detect such issues in other IaC languages. Finally, to simplify practitioners’ adoption of our approach, we aim to enhance the semantic representation of Ansible tasks to overcome the limitation of training a model per Ansible module.

## Footnotes

1 https://github.com/nboro/FindICI

2 Ansible Best Practices: https://docs.ansible.com/ansible/2.8/user∖_guide/playbooks∖_best∖_practices.html

3 http://www.nltk.org/

4 https://www.tensorflow.org/

5 https://keras.io/

6 https://github.com/PyGithub/PyGithub

7 https://github.com/nboro/FindICI

8 https://www.sodalite.eu/

9 Given the number of classifiers and hyper-parameters, we preferred reporting the latter in the online appendix.
